# An opportunity for global antimicrobial stewardship research: Refugee populations

**DOI:** 10.1017/ash.2022.8

**Published:** 2022-02-14

**Authors:** Payal K. Patel, Preeti Mehrotra, Joseph B. Ladines-Lim

**Affiliations:** 1 Division of Infectious Diseases, VA Ann Arbor Healthcare System, Ann Arbor, Michigan; 2 University of Michigan, Michigan Medicine, Ann Arbor, Michigan; 3 Silverman Institute for Health Care Quality and Safety, Boston, Massachusetts; 4 Division of Infectious Diseases, Beth Israel Deaconess Medical Center, Boston, Massachusetts; 5 Departments of Internal Medicine and Pediatrics, University of Michigan, Michigan Medicine, Ann Arbor, Michigan

**Keywords:** antimicrobial stewardship, refugees, infection control and prevention, global health, public health, multidrug-resistant organisms

## Abstract

Antimicrobial resistance is a well-known global health threat that has higher prevalence in the refugee population. Although guidance has been provided by the World Health Organization and Centers for Disease Control and Prevention on implementing antimicrobial stewardship in lower- and middle-income countries, as well as by the United Nations Refugee Agency on other infection prevention and control efforts, no specific guidance exists for implementation of stewardship in this population. We highlight challenges specific to this population, review recent studies of interest within this space, and propose a research agenda to help move stewardship forward in the refugee population. We advocate for the importance of this issue, particularly given recent current events of geopolitical volatility that render this population more vulnerable, in the setting of its already well-known numerous health challenges.

Antimicrobial resistance (AMR) is a well-known global health threat that has been documented with higher prevalence within the refugee population in numerous observational and cohort studies. Such studies have mostly been performed or led by authors from high-income countries.^1–5^ Antimicrobial stewardship programs (ASPs), one of the cornerstones of the Global Action Plan on AMR outlined by the World Health Organization (WHO), have been well studied in resource-rich and resource-limited countries but are less described in refugee populations.^6–10^

## Burden, barriers, and gaps

Although reviews of AMR within refugees have broadly recommended focused screening and use of transmission-based precautions, little else has been detailed on best practices for infection control and prevention within this population, and even less has been considered regarding ASP implementation. Unique challenges exist for ASP implementation within lower- and middle-income countries (LMICs), including political instability, lack of specific governance policies and guidelines, inadequate funding and infrastructure, limited diagnostic and surveillance systems including microbiology laboratories, limited antimicrobial access, inadequate water, sanitation and hygiene (WASH) measures, lack of qualified personnel and training programs, and high infectious disease burden.^6,9,11,12^ Furthermore, there is a high prevalence of inappropriate antimicrobial use in LMICs for numerous reasons including inadequate education on appropriate use of such agents, easier access to these agents from pharmacies and social contacts as opposed to primary healthcare facilities, and lack of enforced regulations on antimicrobial use, among others.^13^ These barriers are most likely applicable to refugee settings because most of this population worldwide resides in LMICs.^14^ The effectiveness of many interventions within LMICs has also been difficult to evaluate due to differences in intervention and outcome evaluations, underinvestment in surveillance capacity, as well as limited quality of related studies.^10^ The WHO and Centers for Disease Control and Prevention (CDC) have published guidelines to assist with ASP development in LMICs, but no specific guidance has been issued on special considerations for refugees.^15,16^ Indeed, there remains lack of guidelines or a common framework from organizations such as the WHO and Médicins Sans Frontières (MSF) and very limited data overall on general medical care of refugees, who reside in settings ranging from self-settled camps to collective centers^17–19^ and who face an infectious disease burden that varies widely with migrant group, ranging from tuberculosis to measles to parasitic infections.^20–22^ We summarize existing knowledge gaps, associated challenges, and suggested solutions in Table 1. This population requires attention for the global antimicrobial stewardship research agenda, given these many health challenges described above, such as intractable substandard living conditions, persistent overcrowding, and malnutrition, among others, that render refugees more prone and vulnerable to infection.^23^

**Table 1.** Knowledge Gaps, Associated Challenges, and Proposed Solutions in Antimicrobial Stewardship and Overall Infection Control and Prevention in Refugee Populations

Gaps	Challenges	Solutions
Lack of guidelines or common framework on general care of refugees from countries as well as institutions such as WHO and MSF Little detail on best practices for infection control and prevention in refugee populations No specific guidance on antimicrobial stewardship in refugee populations	Political instability Lack of specific governance policies and guidelines with respect to refugees Inadequate funding and infrastructure Limited diagnostic and surveillance systems for microbiology Limited antimicrobial access Inadequate WASH measures Lack of qualified personnel and training programs High infectious disease burden High prevalence of inappropriate antimicrobial use	Increased research efforts to inform ASP implementation in refugee population Strengthening of already existing IPC, WASH, and antimicrobial stewardship efforts, particularly in LMICs Application of lessons learned from IPC and WASH to guide agenda setting in antimicrobial stewardship research Advocacy of publication of and adherence to guidelines for ASP at institutional and national level

Note. WHO, World Health Organization; MSF, Médicins Sans Frontières; ASP, antimicrobial stewardship program; WASH, water, sanitation and hygiene; IPC, infection control and prevention; LMICs, lower- and middle-income countries.

## Existing evidence

Existing studies of the refugee population highlight useful information for ASP development in their respective regions and could be used to direct a framework for research on this issue. A cross-sectional study in a rural district in Uganda that hosts refugees from South Sudan and the Democratic Republic of Congo evaluated antibiotic prescribing practices, finding numerous instances of noncompliance with national guidelines, including prescriptions for conditions without any indication for antibiotics such as malaria, viral infection, and noninfectious entities like dysmenorrhea.^24^ The study suggested several potential root causes, such as inadequate training and lack of diagnostic capacity.^24^ Another study at the Thailand-Myanmar border found that in a population largely comprised of migrants and refugees, combined point-of-care testing of urinary tract infections (urine dipstick and microscopy together) improved appropriate antimicrobial prescribing, showing the use of only one test (urine dipstick or urine microscopy alone versus both tests combined when compared to urine culture as the gold standard) to be ineffective in the setting of high prevalence of multidrug resistance in the area.^25^ A qualitative, cross-sectional study in which Palestinian refugees at Jordanian refugee camps were interviewed revealed high prevalence of inappropriate antibiotic use, poor knowledge base of antibiotic side effects and resistance, and long wait times to see providers, preventing refugees from seeking medical advice.^26^ Most inappropriate use was attributed to over-the-counter acquisition of antibiotics without a prescription and self-medication at home or the use of leftover antibiotics from social contacts.^26^ A survey via interview and chart review of pediatric patients within the Rohingya refugee population receiving care at free monthly clinics showed that nearly three-quarters of antimicrobials were not appropriately prescribed.^27^ The majority of this was due to lack of documented diagnosis for antibiotic indication due to lack of time or lack of availability of the indicated antibiotic resulting in prescription of a different agent.^27^ Like much of antimicrobial stewardship, local characteristics will influence ASP outcomes and implementation, but regional studies of refugee populations could be impactful.

## Research need

**Fig. 1.**
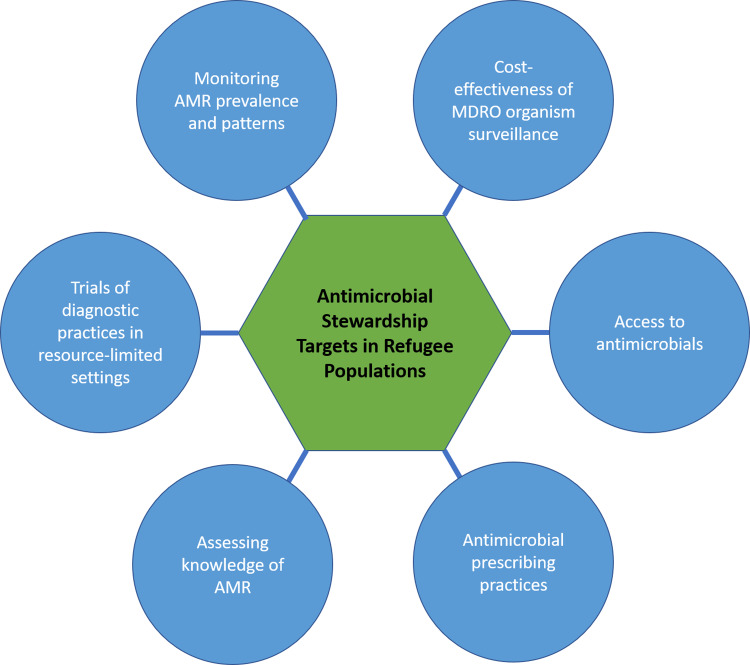
Proposed lines of inquiry in antimicrobial stewardship in refugee populations.

Although it is just a tiny fraction of the published literature on refugee health, the existing studies on antibiotic use in this population lay groundwork for future research to inform ASP implementation in this population. In Figure 1, we describe potential fields of inquiry including monitoring of AMR prevalence and patterns in this population; effectiveness of multidrug-resistant organism surveillance; antimicrobial availability and access; antimicrobial prescribing practices, attitudes, and knowledge of both providers and refugees with respect to AMR; and assessment of diagnostic practices in resource-limited settings. This partially overlaps with the core elements of ASPs described by the WHO and CDC, which should also be pursued in full at the national level.^28^ Notably, efforts toward antimicrobial stewardship in LMICs are currently underway in numerous forms, including evaluations of hospital antibiotic stewardship programs, expansion of AMR surveillance networks, community- and education-based interventions, use of interdisciplinary teams, and international collaborations, among others.^10,29–40^ Organizations such as MSF also have numerous active projects in this space, including stewardship implementation and expansion of diagnostic capacity, as well as clinical trials, such as a recent study revealing lack of efficacy of routine antibiotic use for uncomplicated severe acute malnutrition in Niger and another recent study of the epidemiology of drug-resistant bloodstream infections in burn patients in Iraq.^41–43^ These efforts in LMICs could serve as a starting point for adaptation in the refugee population.

Considerations should also be made for differences in living situation among refugee populations. Those residing in congregate settings such as camps, such as the Rohingya in Cox’s Bazar,^44^ may have fluctuating population totals and may be subject to geopolitical instability. Conflict regions, such as certain Arab countries, particularly exacerbate the problem for both refugees and home populations alike.^45^ Heavy metal use is an additional factor contributing to AMR that is not readily addressed by stewardship programs.^46^ Even those refugees resettled in urban centers in more stable host countries may face their own challenges in integration.^47^

Much work remains to be done in integrating ASPs in healthcare settings around the world. This issue is especially critical in refugee populations. Although they have garnered an increasing amount of literature in recent years,^48^ including documentation of increased AMR prevalence, little attention has been given to the implementation challenges in such settings. Preliminary published data and lessons learned from WASH and infection prevention and control efforts should guide future agenda setting. Technical guidance from the United Nations Refugee Agency (UNHR) offers direction on a range of infection prevention and control topics, including disinfection and sterilization as well as COVID-19 WASH efforts.^49,50^ Creating similar and complementary evidence-based ASP guidance for practitioners is critical to moving the global AMR agenda forward.

Recent events, such as the withdrawal of United States military forces from Afghanistan and the arrival of Haitian asylum seekers at the United States southern border in the wake of the Haitian earthquake and presidential assassination further highlight the geopolitical volatility that places these populations at high risk. These populations already show higher prevalence rates of multidrug-resistant organism carriage.^1–5^ Without such targeted efforts, progress of refugee health efforts will stall, risking loss of the progress of ASP efforts not only in this population but in LMICs worldwide.

## References

[1] Maltezou HC , Theodoridou M , Daikos GL. Antimicrobial resistance and the current refugee crisis. J Glob Antimicrob Resist 2017;10:75–79. 28673700 10.1016/j.jgar.2017.03.013

[2] Maltezou HC , Elhadad D , Glikman D. Monitoring and managing antibiotic resistance in refugee children. Expert Rev Anti Infect Ther 2017;15:1015–1025. 29027495 10.1080/14787210.2017.1392853

[3] Nellums LB , Thompson H , Holmes A , et al. Antimicrobial resistance among migrants in Europe: a systematic review and meta-analysis. Lancet Infect Dis 2018;18:796–811. 29779917 10.1016/S1473-3099(18)30219-6 PMC6032478

[4] Heudorf U , Albert-Braun S , Hunfeld K-P , et al. Multidrug-resistant organisms in refugees: prevalences and impact on infection control in hospitals. GMS Hyg Infect Control 2016;11:Doc16. 27579250 10.3205/dgkh000276 PMC4987489

[5] de Smalen AW , Ghorab H , Abd El Ghany M , Hill-Cawthorne GA. Refugees and antimicrobial resistance: a systematic review. Travel Med Infect Dis 2017;15:23–28. 27919742 10.1016/j.tmaid.2016.12.001

[6] Hijazi K , Joshi C , Gould IM. Challenges and opportunities for antimicrobial stewardship in resource-rich and resource-limited countries. Expert Rev Anti Infect Ther 2019;17:621–634. 31282277 10.1080/14787210.2019.1640602

[7] Pierce J , Apisarnthanarak A , Schellack N , et al. Global antimicrobial stewardship with a focus on low- and middle-income countries. Int J Infect Dis 2020;96:621–629. 32505875 10.1016/j.ijid.2020.05.126 PMC7271868

[8] Pierce J , Apisarnthanarak A , Schellack N , et al. Global antimicrobial stewardship with a focus on low- and middle-income countries. Int J Infect Dis 2020;96:621–629. 32505875 10.1016/j.ijid.2020.05.126 PMC7271868

[9] Dapás JI , Quirós RE. Antimicrobial stewardship in low- and middle-income countries. Curr Treat Options Infect Dis 2018;10:17–27.

[10] van Dijck C , Vlieghe E , Cox JA. Antibiotic stewardship interventions in hospitals in low-and middle-income countries: a systematic review. Bull World Health Org 2018;96:266–280. 29695883 10.2471/BLT.17.203448 PMC5872012

[11] Cox JA , Vlieghe E , Mendelson M , et al. Antibiotic stewardship in low- and middle-income countries: the same but different? Clin Microbiol Infect 2017;23:812–818. 28712667 10.1016/j.cmi.2017.07.010

[12] Tiong JJL , Loo JSE , Mai C-W. Global Antimicrobial stewardship: a closer look at the formidable implementation challenges. Front Microbiol 2016;7:1860. 27899924 10.3389/fmicb.2016.01860 PMC5110512

[13] Aslam A , Gajdács M , Zin CS , et al. Evidence of the practice of self-medication with antibiotics among the lay public in low- and middle-income countries: a scoping review. Antibiot 2020;9:597. 10.3390/antibiotics9090597 PMC7558641 32932630

[14] United Nations High Commissioner for Refugees. Refugee data finder. United Nations website. https://www.unhcr.org/refugee-statistics/download/. Accessed January 18, 2022.

[15] World Health Organization. Antimicrobial Stewardship Programmes in Health Facilities in Low- and Middle-Income Countries: A WHO Practical Toolkit. Geneva: WHO; 2019. 10.1093/jacamr/dlz072 PMC8210188 34222945

[16] Centers for Disease Control. The Core Elements of Human Antibiotic Stewardship Programs in Resource-Limited Settings: National and Hospital Levels. Atlanta: CDC; 2018.

[17] Blundell H , Milligan R , Norris SL , Garner P. WHO guidance for refugees in camps: systematic review. BMJ Open 2019;9:e027094. 10.1136/bmjopen-2018-027094 PMC6731884 31488468

[18] Lebano A , Hamed S , Bradby H , et al. Migrants’ and refugees’ health status and healthcare in Europe: a scoping literature review. BMC Public Health 2020;20:1–22. 32605605 10.1186/s12889-020-08749-8 PMC7329528

[19] Piselli P , Samuilova M , Bozorgmehr K , et al. Infectious-disease screening and vaccination for refugees and asylum seekers entering Europe in 2015–16: a scoping study of six European Union countries. J Refug Stud 2019;32 special_issue_1:i92–i104. 10.1016/j.healthpol.2018.04.003 29673804

[20] Eiset AH , Wejse C. Review of infectious diseases in refugees and asylum seekers—current status and going forward. Public Health Rev 2017;38:1–16. 29450094 10.1186/s40985-017-0065-4 PMC5810046

[21] Janda A , Eder K , Fressle R , et al. Comprehensive infectious disease screening in a cohort of unaccompanied refugee minors in Germany from 2016 to 2017: a cross-sectional study. PLOS Med 2020;17:e1003076. 32231358 10.1371/journal.pmed.1003076 PMC7108686

[22] Altare C , Kahi V , Ngwa M , et al. Infectious disease epidemics in refugee camps: a retrospective analysis of UNHCR data (2009-2017). J Glob Heal Rep. 2019;3:e2019064.

[23] Allotey P , Reidpath D , eds. The Health of Refugees: Public Health Perspectives from Crisis to Settlement, 2 *nd edition.* New York; Oxford University Press; 2019.

[24] Bonniface M , Nambatya W , Rajab K. An evaluation of antibiotic prescribing practices in a rural refugee settlement district in Uganda. Antibiotics 2021;10:172. 33572240 10.3390/antibiotics10020172 PMC7915286

[25] Chalmers L , Cross J , Chu CS , et al. The role of point-of-care tests in antibiotic stewardship for urinary tract infections in a resource-limited setting on the Thailand–Myanmar border. Trop Med Int Heal 2015;20:1281–1289. 10.1111/tmi.12541 PMC4758398 25963224

[26] Al Baz M , Law MR , Saadeh R. Antibiotics use among Palestine refugees attending UNRWA primary healthcare centers in Jordan—a cross-sectional study. Travel Med Infect Dis 2018;22:25–29. 29458088 10.1016/j.tmaid.2018.02.004

[27] Tahir ARM , Ee XW , Rashid AA , Yahaya AY bin, Devaraj NK . The proportion of infectious disease cases, its associated factors, and the appropriateness of antimicrobial prescription among rohingya refugee pediatric patients in IMARET mobile clinics. J Immigr Minor Heal 2021;23:1159–1169. 10.1007/s10903-021-01150-6 33543426

[28] World Health Organization. WHO Policy Guidance on Integrated Antimicrobial Stewardship Activities. Geneva: WHO; 2021.

[29] Gandra S , Alvarez-Uria G , Turner P , Joshi J , Limmathurotsakul D , van Doorn HR. Antimicrobial resistance surveillance in low-and middle-income countries: progress and challenges in eight South Asian and Southeast Asian countries. Clin Microbiol Rev 2020;33(3):1–29. 10.1128/CMR.00048-19 PMC7289787 32522747

[30] Cooper L , Sneddon J , Afriyie DK , et al. Supporting global antimicrobial stewardship: antibiotic prophylaxis for the prevention of surgical site infection in low- and middle-income countries (LMICs): a scoping review and meta-analysis. JAC Antimicrobial Resist 2020;2(3):dlaa070. 10.1093/jacamr/dlaa070 PMC8210156 34223026

[31] Mendelson M , Røttingen JA , Gopinathan U , et al. Maximising access to achieve appropriate human antimicrobial use in low-income and middle-income countries. Lancet 2016;387:188–198. 26603919 10.1016/S0140-6736(15)00547-4

[32] Charani E , Smith I , Skodvin B , et al. Investigating the cultural and contextual determinants of antimicrobial stewardship programmes across low-, middle- and high-income countries—a qualitative study. PLoS One 2019;14(1):e0209847. 30650099 10.1371/journal.pone.0209847 PMC6335060

[33] Dondorp AM , Limmathurotsakul D , Ashley EA. What’s wrong in the control of antimicrobial resistance in critically ill patients from low- and middle-income countries? Intensive Care Med 2018;44:79–82. 28409205 10.1007/s00134-017-4795-z PMC5770509

[34] Howard P , Pulcini C , Levy Hara G , et al. An international cross-sectional survey of antimicrobial stewardship programmes in hospitals. J Antimicrob Chemother 2014;70:1245–1255. 25527272 10.1093/jac/dku497

[35] Prentiss T , Weisberg K , John Zervos C , Address J. Building capacity in infection prevention and antimicrobial stewardship in low- and middle-income countries: the role of partnerships inter-countries. Curr Treat Options Infect 2018;10:7–16.

[36] Pauwels I , Versporten A , Vermeulen H , Vlieghe E , Goossens H. Assessing the impact of the Global Point Prevalence Survey of Antimicrobial Consumption and Resistance (Global-PPS) on hospital antimicrobial stewardship programmes: results of a worldwide survey. Antimicrob Resist Infect Control 2021;10:1–12. 34583775 10.1186/s13756-021-01010-w PMC8478001

[37] Mendelson M , Dar OA , Hoffman SJ , Laxminarayan R , Mpundu MM , Røttingen JA. A global antimicrobial conservation fund for low- and middle-income countries. Int J Infect Dis 2016;51:70–72. 27647658 10.1016/j.ijid.2016.09.016

[38] Sulis G , Sayood S , Gandra S. Antimicrobial resistance in low- and middle-income countries: current status and future directions. 2021. doi: 10.1080/14787210.2021.1951705. 10.1080/14787210.2021.1951705 34225545

[39] Foxlee ND , Townell N , Heney C , McIver L , Lau CL. Strategies used for implementing and promoting adherence to antibiotic guidelines in low- and lower–middle-income countries: a systematic review. Trop Med Infect Dis 2021;6:166. 34564550 10.3390/tropicalmed6030166 PMC8482147

[40] Lam TT , Dang DA , Tran HH , et al. What are the most effective community-based antimicrobial stewardship interventions in low- and middle-income countries? A narrative review. J Antimicrob Chemother 2021;76:1117–1129. 33491090 10.1093/jac/dkaa556

[41] Antibiotic resistance. Médicins Sans Frontières website. https://www.msf.org/antibiotic-resistance. Accessed January 18, 2022.

[42] Ronat JB , Kakol J , Khoury MN , et al. Highly drug-resistant pathogens implicated in burn-associated bacteremia in an Iraqi burn care unit. PLoS One 2014;9(8):e101017. 25111170 10.1371/journal.pone.0101017 PMC4128596

[43] Isanaka S , Langendorf C , Berthé F , et al. Routine amoxicillin for uncomplicated severe acute malnutrition in children. N Engl J Med 2016;374:444–453. 26840134 10.1056/NEJMoa1507024

[44] Bhatia A , Mahmud A , Fuller A , et al. The Rohingya in Cox’s Bazar: when the stateless seek refuge. Health Hum Rights 2018;20:105. 30568406 PMC6293360

[45] Reffat N , Khoshnood K , Dembry L-M. Evidence-based interventions for antimicrobial resistance in conflict-afflicted Arab countries. In: Laher I , editor. Handbook of Healthcare in the Arab World. New York: Springer; 2021:1–25.

[46] Bazzi W , Abou Fayad AG , Nasser A , et al. Heavy metal toxicity in armed conflicts potentiates AMR in *A. baumannii* by selecting for antibiotic and heavy metal coresistance mechanisms. Front Microbiol 2020;0:68. 10.3389/fmicb.2020.00068 PMC7008767 32117111

[47] Donato KM , Ferris E. Refugee integration in Canada, Europe, and the United States: perspectives from research. Ann Acad Pol Sci Soc 2020;690:7–35.

[48] Sweileh WM. Bibliometric analysis of medicine—related publications on refugees, asylum seekers, and internally displaced people: 2000–2015. BMC Int Health Hum Rights 2017;17:7. 28320410 10.1186/s12914-017-0116-4 PMC5360014

[49] United Nations High Commissioner for Refugees. COVID-19 WASH resources. United Nations website. https://wash.unhcr.org/covid-19-resources/. Published 2020. Accessed January 18, 2022.

[50] United Nations High Commissioner for Refugees. Condensed guidance on disinfection solutions. United Nations website. https://www.unhcr.org/en-us/publications/brochures/5f0316b94/condensed-guidance-disinfection-solutions.html. Published 2020. Accessed January 18, 2022.

